# Methodology and reporting characteristics of studies using interrupted time series design in healthcare

**DOI:** 10.1186/s12874-019-0777-x

**Published:** 2019-07-04

**Authors:** Jemma Hudson, Shona Fielding, Craig R. Ramsay

**Affiliations:** 1 Health Services Research Unit, University of Aberdeen, Health Sciences Building, Foresterhill, Aberdeen, AB25 2ZD UK; 2 Medical Statistics Team, Institute of Applied Health Sciences, University of Aberdeen, Foresterhill, Aberdeen, AB25 2ZD UK

**Keywords:** Interrupted time series, Quasi-experimental, Healthcare interventions

## Abstract

**Background:**

Randomised controlled trials (RCTs) are considered the gold standard when evaluating the causal effects of healthcare interventions. When RCTs cannot be used (e.g. ethically difficult), the interrupted time series (ITS) design is a possible alternative. ITS is one of the strongest quasi-experimental designs. The aim of this methodological study was to describe how ITS designs were being used, the design characteristics, and reporting in the healthcare setting.

**Methods:**

We searched MEDLINE for reports of ITS designs published in 2015 which had a minimum of two data points collected pre-intervention and one post-intervention. There was no restriction on participants, language of study, or type of outcome. Data were summarised using appropriate summary statistics.

**Results:**

One hundred and sixteen studies were included in the study. Interventions evaluated were mainly programs 41 (35%) and policies 32 (28%). Data were usually collected at monthly intervals, 74 (64%). Of the 115 studies that reported an analysis, the most common method was segmented regression (78%), 55% considered autocorrelation, and only seven reported a sample size calculation. Estimation of intervention effects were reported as change in slope (84%) and change in level (70%) and 21% reported long-term change in levels.

**Conclusions:**

This methodological study identified problems in the reporting of design features and results of ITS studies, and highlights the need for future work in the development of formal reporting guidelines and methodological work.

**Electronic supplementary material:**

The online version of this article (10.1186/s12874-019-0777-x) contains supplementary material, which is available to authorized users.

## Background

Rigorous evaluations are the cornerstone of evidence-based healthcare. The gold standard for evaluating the causal effect of an intervention is a randomised controlled trial (RCT). However, RCTs do have limitations, they can be costly, unethical or impractical to conduct [1–3]. Therefore, researchers must consider alternative designs to evaluate interventions and quasi-experimental studies are one possible solution [4]. Quasi-experimental studies do not use randomisation and may use both pre-and post-intervention data. Interrupted time series (ITS) is considered one of the strongest quasi-experimental designs [4]. 

In an ITS design, data are collected at multiple and equally spaced time points (e.g. weekly, monthly, or yearly) before and after an intervention. Knowing the exact time when an intervention occurs is an important feature. The main objective of an ITS is to examine whether the data pattern observed post-intervention is different to that observed pre-intervention. There are a range of effect estimates to describe the impact of the intervention. For example, a change in level corresponds to the difference in the time point of interest to the predicted pre-intervention trend or a change in slope which is the difference between the post-and pre-intervention slopes [5]. 

When designing an ITS design and analysing the data there are important characteristics that need to be considered, these include (1) autocorrelation, whereby data collected closely together are correlated with each other, (2) nonstationary or secular trend, which is where the data are increasing or decreasing over time irrespective of any intervention, (3) seasonality or cyclic patterns, (4) outliers, (5) other interventions (interruptions) occurring in the data series, and (6) sample size.

Three systematic reviews [5–7] have looked at ITS designs but all focussed on specific, narrow areas. Polus et al [6] focused on pre-specified intervention types (for example behavioural/educational, clinical, environmental, health policy, and health systems) and only included 16 studies from Cochrane Effective Practice and Organisation of Care (EPOC) reviews. The key findings were ITS terminology is used for a variety of study designs and when an ITS design is used the intervention is at the organisational level. Jandoc et al [7] included 220 studies but only focused on drug utilisation. They found ITS designs increasingly being used but reporting standards varied. Ramsay et al [5] concentrated on mass media interventions, included 58 studies, concentrated on methodology quality and concluded that in most cases there was poor reporting of study design. To our knowledge, no study has assessed the breadth of use of ITS designs across healthcare settings.

The aim of this methodological study was to identify a cohort of ITS designs across all healthcare settings and to describe how ITS are being used, what design characteristics are considered, and how they are reported.

## Methods

This methodological study was conducted according to a prespecified study protocol [8]. 

### Inclusion and exclusion criteria

We included ITS with a minimum of two data points pre and one post-intervention that assessed a health or healthcare intervention (e.g. programs, policies, or educational interventions). Systematic reviews, meta-analysis, RCTs, ITS designs with a control group or studies that did not use an ITS analysis were excluded. There were no restrictions on participants, language of study, or the type of outcome.

### Search strategy

We searched MEDLINE(R) and Epub Ahead of Print, In-Process & Other Non-Indexed Citations and Daily) in October 2016 for ITS designs published in 2015. The search strategy is in Additional file 1.

JH screened titles and abstracts identified by the search for inclusion. CR and SF double assessed 10% of the titles and abstracts and if there were no disagreements then JH would proceed to single screening. Full-text copies for all the potential studies were obtained and assessed for inclusion by JH with CR and SF double assessing 10%.

### Data extraction and analysis

A data extraction form was developed and piloted on three randomly selected papers by all authors. JH recorded the relevant details of all included studies onto a data extraction form. Two authors (CR and SF) independently assessed 10% of randomly selected studies and if there were no disagreements then JH would proceed to data extract the remaining studies. Data extracted from the studies consisted of: definition of study design (e.g. ITS, before-and-after), country of study, study objectives (population, intervention and the outcomes of interest), type and level of intervention, participants, data source, type of outcome, the number of data points collected pre-and post-intervention and the frequency, study methodology characteristics, estimations of intervention effects and the reporting details of the abstract and discussion. We based data extraction on the primary outcome and if no defined primary outcome was reported, we used the first reported outcome. We summarised data using descriptive statistics (numbers and percentages or median, 25th, and 75th centile).

As a methodological study, risk of bias assessment was not performed on individual studies.

## Results

**Fig. 1:**
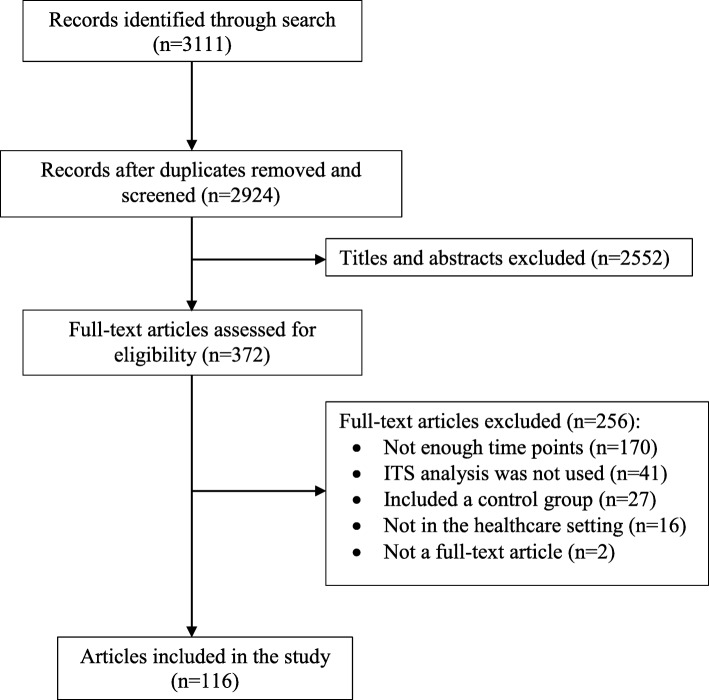
PRISMA diagram

The search strategy identified 3111 title and abstracts (Fig. 1). After removing duplicates (187) and excluding 2552 titles and abstracts that did not meet the inclusion criteria, 372 full-text studies were assessed for eligibility. Of these, 256 articles were excluded with the majority (170) having too few time points. A total of 116 articles were included in the study and the list of included studies is provided in Additional file 2. As there were no disagreements in the screening and data extraction, CR and SF only double assessed 10%.

In the abstract of the included studies, the intervention was clearly defined in 110 (95%) of studies (Table 1). The method of analysis was reported in 57% of the studies and the number of pre-and post-intervention data points were stated in 29% and 28% of studies respectively. The main results were reported in three quarters of the studies.

**Table 1:** ITS study characteristics described in the scientific abstract

	*N* = 116
Method of analysis given	66 (57)
Number of pre-intervention points stated	34 (29)
Number of post-intervention points stated	33 (28)
Main results reported	85 (73)

Values are *n* (%)

Characteristics of the included studies are presented in Table 2. Seventy four (64%) studies provided a study definition in at least the title, abstract, or the main paper. Of these 9 (12%) provided a definition in the title, abstract, and main paper with only two consistently giving the same definition. Examples of study definitions used include ITS, quasi-experimental, time series, observational, cohort, and cross-sectional study. The majority of studies were from the US (34%), the UK (16%), Asia (16%), and Europe (12%). The type of intervention varied, the most common being programs (35%) and policies (28%). Interventions were mainly at the hospital level (63%), aimed at health professionals (79%), and over half of the data came from hospitals (55%). Data were mainly collected at monthly intervals (64%), and the number of data points collected pre-and post-intervention was mostly the same within any given study with a median ratio of one (25th, 75th centile 1–2). Additionally, Study objectives were clearly defined in 85 (73%) of studies and 17 (15%) gave a rationale for using an ITS design. The majority of the studies investigated one intervention (78%), 15% studied two and 8% studied three or more. Only 30 (26%) specified a primary and/or secondary outcome and 107 (92%) clearly defined when the intervention occurred. Eighty-three (72%) gave a funding source statement, 7 (6%) studies referred to a study protocol, and only one study was non-English (Spanish).

**Table 2:** Study characteristics of included ITS

	N = 116
Study definition provided in at least title, abstract or main paper	74 (64)
Title, abstract, and main paper	9 (12)
Abstract and main paper	16 (22)
Title and main paper	17 (23)
Title and abstract	1 (1)
Title	5 (7)
Abstract	7 (9)
Main paper	19 (26)
Country of study	
USA	39 (34)
UK	18 (16)
Asia	18 (16)
Europe	14 (12)
Canada	11 (9)
Australia	7 (6)
Africa	6 (5)
Middle East	2 (2)
Panama	1 (1)
Type of intervention	
Programs (e.g. multifaceted)	41 (35)
Policy (e.g. regulatory)	32 (28)
Health systems	25 (22)
Guidelines	19 (16)
Financial	19 (16)
Behavioural	15 (13)
Sales and dispensing	1 (1)
Level of intervention	
Hospital	73 (63)
Hospital department	22 (19)
Individual	17 (15)
GP practices	3 (3)
Pharmacy	1 (1)
Participants	
Health professional	92 (79)
Disease	30 (26)
Occupational	3 (3)
Population	1 (1)
Data source	
Hospital data	64 (55)
Health records	27 (23)
National data	26 (22)
Insurance data	11 (9)
Other	3 (3)
Type of outcome	
Continuous	66 (57)
Rate	38 (33)
Binary	11 (9)
Count	1 (1)
Frequency	
Monthly	74 (64)
Quarterly	23 (20)
Yearly	14 (12)
Weekly	2 (2)
Other^a^	3 (3)
Number of data points - median (25th,75th centile)	
Pre-intervention	18 (12–32)
Post-intervention	19 (12–34)
Ratio of pre/ post-intervention data points	1 (1–2)
Transition period (*n* = 20)	3 (1–9)
Other data points that were accounted for (*n* = 3)	2 (2–8)

Values are *n* (%) unless otherwise stated. ^a^ 90 day periods; five times over two weeks before and after; daily

Abbreviations: *US* United States, *UK* United Kingdom

Characteristics of the methodology used in each study showed all but one study provided a description of the analysis, with segmented regression analysis being used in 90 (78%) studies (Table 3). Autocorrelation was considered in 63 (55%) studies, of these only 40 (63%) reported any formal testing. Only 9 (8%) studies considered nonstationary and seasonality was considered in 28 (24%).

**Table 3:** Methodology characteristics in included ITS

	*N* = 116
Description of the analysis	115 (99)
Segmented regression	90 (78)
ARIMA model	15 (13)
Generalised estimating equations	7 (6)
Change-point analysis	2 (2)
Mixed model	1 (1)
Autocorrelation was considered	63/115 (55)
Method used to test for autocorrelation^a^	*N* = 63
Durbin Watson	22 (35)
Autocorrelation function	13 (21)
Partial autocorrelation function	11 (17)
Ljung-Box	3 (5)
Examination of residuals	2 (3)
Portmanteau tests	2 (3)
Autocorrelation probability	1 (2)
No test performed	23 (37)
Autocorrelation present if a test was performed	*N* = 40
No	12 (30)
Yes	25 (63)
Not stated	3 (8)
Method used to adjust for autocorrelation	*N* = 48
Autoregressive error term	14 (29)
Prais-winsten regression model	8 (17)
Differencing	3 (6)
Newey-west standard errors	2 (4)
Yule-walker regression model	2 (4)
Did not specify	19 (40)
Order of autocorrelation	N = 48
1	8 (17)
2	3 (6)
3	2 (4)
4	1 (2)
5	1 (2)
Did not specify	33 (69)
Nonstationary was considered	9/115 (8)
Method used to test for non-stationary	*N* = 9
Dicky-Fuller	5 (56)
ACF and PACF	2 (22)
Significance testing	1 (11)
Not stated	1 (11)
Nonstationary was present if a test was performed	*N* = 8
No	4 (50)
Yes	3 (38)
Not stated	1 (13)
Method used to adjust for nonstationary	N = 3
Differencing	2 (67)
Within the ARIMA model	1 (33)
Seasonality was considered	28/115 (24)
Method used to test for seasonality	*N* = 28
ACF PACF	5 (18)
Regression diagnostic tests	1 (4)
Dicky-Fuller	1 (4)
Just stated a test was performed	4 (14)
No formal test	17 (61)
Seasonality present if a test was performed	*N* = 7
No	6 (86)
Yes	1 (14)
Method used to adjust for seasonality	*N* = 22
Covariate	7 (32)
Seasonal ARIMA	1 (5)
Differencing	1 (5)
Not stated	13 (59)
Sample size description	*N=115*
No	108 (94)
Yes	7 (6)

Values are n (%). Abbreviations: *ARIMA*, Autoregressive integrated moving average; *GEE* generalized estimating equation; *ACF*, autocorrelation function; *PACF* Partial autocorrelation function

Seven (6%) studies provided a sample size description, three gave a discussion only with no formal calculation and four gave a calculation of which one could be reproduced. For these four, the sample size was based on detecting an “importance difference” for two studies, while the remainder determined what effect size and power was detectable based on data they had. However all four based the sample size on comparing differences in proportions pre- and post-intervention.

The unit of analysis was the same as the unit of intervention in 74 (64%) studies and a description of how missing data was handled was reported in 5% of studies. A transition period, a period which allows the intervention to take affect was considered in 17% of studies. Sensitivity analysis was carried out in 20 (17%) of studies. Of these, 16 studies did another form of analysis (e.g. adjusted for autocorrelation, seasonality, and covariates), and two considered a transition period.

Data were presented graphically in 109 (94%) of studies. Three (3%) studies reported outliers in their data but only one reanalysed the data with the data point removed. Results of the analysis were reported by 115 (99%) with both relative and absolute figures presented (Table 4). Change in slope (84%) and change in level (70%) were the most common intervention effects reported with 33 (28%) reporting both. Of the studies that reported a change in level, 75 (93%) reported an immediate change while 17 (21%) reported other change in levels, for example, 12 months after the intervention. Only 8% reported confidence intervals (CIs) or standard errors (SEs) along with the relative slope effects, 80% for an immediate change in level effect and 65% for other change in level effects.

**Table 4:** ITS study effect sizes reported

	*N* = 116
Relative effects	
Relative slope	13 (11)
Relative to:	*N* = 13
Baseline trend	8 (62)
Not stated	5 (38)
CI/SE reported	1 (8)
*p*-value reported	2 (15)
Relative level	16 (14)
Relative to:	*N* = 16
Baseline trend	13 (81)
Last pre-intervention data point	1 (6)
Not stated	2 (13)
CI/SE reported	8 (50)
p-value reported	6 (38)
Absolute effects	
Change in slope	97 (84)
CI/SE reported	74/97 (76)
p-value reported	84/97 (87)
Change in level	81 (70)
Immediate	75/81 (93)
CI/SE reported	60/75 (80)
p-value reported	67/75 (89)
Other level effects	17/81 (21)
CI/SE reported	11/17 (65)
p-value reported	8/17 (47)
Other estimates reported	
Intercept	40 (34)
Pre-slope trend	74 (64)
Post-slope trend	15 (13)

Values are *n* (%). Abbreviations: *CI* Confidence interval, *SE* Standard error

Table 5 describes the characteristics of the discussion section of the studies. All studies gave an overall interpretation of results, 113 (97%) studies summarised key results and one study discussed the impact of outliers in their data. Forty five (39%) gave a discussion or statement of whether other co-interventions might have taken place in the study. Weakness and limitations were discussed in 98 (84%) of studies, and 39 (34%) indicated strengths.

**Table 5:** Discussion of findings in ITS studies

	*N* = 116
Key results summarised with reference to objectives	
No	2 (2)
Yes	113 (97)
Some	1 (1)
Discussion of bias	65 (56)
Weaknesses/limitations	98 (84)
Strengths	39 (34)

Values are *n* (%)

## Discussion

To our knowledge this methodological study is the first to show inconsistencies and gaps in the reporting of design features and results of ITS design across a variety of healthcare studies and the first to describe the different ways of reporting effect estimates (e.g. relative slope and level effects and absolute change in slope and level).

Reporting effect estimates is vital to interpret studies, but due to the variety of possible estimates that could be reported, this can cause challenges across multiple studies. For example, a change in level corresponds to the difference in the time point of interest to the predicted pre-intervention trend where a change in slope is the difference between the post- and pre-intervention slopes [5]. The different effect estimates cannot be combined together. Therefore, although most individual studies in this study reported an effect size, the many different ways of reporting make meta-analyses difficult. In addition, none of the included studies based the sample size justification on the effect size of interest, making interpretation of a meaningful difference difficult. For example, the sample size calculation compared differences in proportions pre- and post-intervention, but the effect size was a difference in slope [9]. 

Only 74 (64%) of studies provided a study definition in at least the title, abstract, or the main paper with definitions varying between them and only 29 studies referring to ITS. As ITS studies can be included in systematic reviews a clear and consistent definition is needed. This is so researchers can identify ITS studies to be included in their review and be sure no studies are missed because of the study definition.

This study identified five statistical methods used to analysis ITS data (Table 5), however there has been no research comparing ITS methods to determine which one to use and when. Also, the varying statistical methods can have implications on what effect estimates are reported, which can impact on the interpretation of results and effect the pooling of results for a meta-analysis.

It was not possible to determine whether the statistical analysis performed was appropriate in some cases. Our study found that one study did not provide any description of the analysis and of the 115 that did only 55% considered autocorrelation, 10% non-stationary and 28% seasonality. These figures are slightly lower than the previous reviews of Jandoc et al [7]. (autocorrelation 66%; non-stationary 15%; seasonality 31%), while Polus et al [6]. reported autocorrelation and non-stationary in 31% of studies. For ITS studies these considerations are important as it can cause the results either to be underestimated or overestimated which could affect the overall conclusions of studies.

This study had no restrictions on the type of intervention included, language of report and included 116 studies, therefore the findings are representative of ITS studies that are published. However there were some limitations. Only one year was searched, 2015, but there are no reasons to believe that other years would give a markedly different perspective. We potentially could have missed papers due to the inconsistency in the reporting of the definition of an ITS study as well as not searching grey literature and other databases but we do not see these limitations biasing the representativeness of ITS studies.

## Conclusions

Currently reporting is poor. Jandoc et al [7] provided reporting recommendations of ITS studies, however our study has identified gaps in reporting which are not included. In the statistical methods section of the study, a description of a sample size calculation or a justification should be provided and if present, how missing data was handled. For reporting of effect sizes, there needs to be consistency in what should definitely be reported along with confidence intervals. Also, the study design should be indicated in the main paper in addition to the title and abstract. Therefore, there is a need to provide clearer guidelines for reporting standards through consensus approaches such as a consensus meeting or a Delphi study. In additional to poor reporting of ITS studies, this study highlighted that there are numerous ways of analysing ITS studies. This can make interpretation of results difficult, for example presenting effect sizes as either relative or absolute. This illustrates the need to assess methodological strengths and weaknesses of current ITS analysis methods.

## Additional files

Additional file 1: Search strategy (DOCX 14 kb)

Additional file 2: References for included studies (DOCX 29 kb)

## Data Availability

The data appeared in this study are already publicly available in the literature.

## Abbreviations

CI: Confidence Interval
EPOC: Effective Practice and Organisation of Care
ITS: Interrupted Time Series
RCT: Randomised Controlled Trial
SE: Standard Error
