# Supplementary material for: Methodology and reporting characteristics of studies using interrupted time series design in healthcare
Source: BMC Med Res Methodol. 2019 Jul 4;19:137. doi: 10.1186/s12874-019-0777-x (PMC6609377; doi:10.1186/s12874-019-0777-x)
Supplement: Supplementary file 1 — Search strategy (DOCX 14 kb) [file 12874_2019_777_MOESM1_ESM.docx]

**Search strategy**

1. interrupted time series.tw,kw.
2. (pre adj1 post).tw.
3. (segmented adj3 regression).tw,kw.
4. quasi experiment$.tw,kw.
5. (before adj1 after).tw,kw.
6. arima.tw,kw.
7. (trend adj3 analys?s).tw,kw.
8. (longitudinal adj3 chang$).tw,kw.
9. autoregressive integrated moving average.tw,kw.
10. or/1-9
11. limit 10 to yr='2015'
12. randomi$ control$ trial$.ti,tw.
13. meta analys$.ti,kw.
14. limit 11 to "review articles"
15. 11 not (12 or 13 or 14)
