# Supplementary material for: Methodology and reporting characteristics of studies using interrupted time series design in healthcare
Source: BMC Med Res Methodol. 2019 Jul 4;19:137. doi: 10.1186/s12874-019-0777-x (PMC6609377; doi:10.1186/s12874-019-0777-x)
Supplement: Supplementary file 2 — References for included studies (DOCX 29 kb) [file 12874_2019_777_MOESM2_ESM.docx]

**References for included studies**

1. Aljeesh YI, Alkariri N, Abusalem S, Myers JA, Alaloul F. Staff-Developed Infection Prevention Program Decreases Health Care–Associated Infection Rates in Pediatric Critical Care. J Nurs Care Qual 2015;30(1).

2. Baker A, Chen L, Elliott RA, Godman B. The impact of the ‘Better Care Better Value’ prescribing policy on the utilisation of angiotensin-converting enzyme inhibitors and angiotensin receptor blockers for treating hypertension in the UK primary care setting: longitudinal quasi-experimental design. BMC Health Services Research 2015;15(1):367.

3. Balasubramanian D, Prinja S, Aggarwal AK. Effect of User Charges on Secondary Level Surgical Care Utilization and Out-of-Pocket Expenditures in Haryana State, India. PLoS ONE 2015;10(5):e0125202.

4. Bao L, Peng R, Wang Y, Ma R, Ren X, Meng W, et al. Significant Reduction of Antibiotic Consumption and Patients’ Costs after an Action Plan in China, 2010–2014. PLoS ONE 2015;10(3):e0118868.

5. Berkowitz SA, Percac-Lima S, Ashburner JM, Chang Y, Zai AH, He W, et al. Building Equity Improvement into Quality Improvement: Reducing Socioeconomic Disparities in Colorectal Cancer Screening as Part of Population Health Management. Journal of General Internal Medicine 2015;30(7):942-949.

6. Bhindi B, Mamdani M, Kulkarni GS, Finelli A, Hamilton RJ, Trachtenberg J, et al. Impact of the U.S. Preventive Services Task Force Recommendations against Prostate Specific Antigen Screening on Prostate Biopsy and Cancer Detection Rates. J Urol 2015;193(5):1519-1524.

7. Bijlsma MJ, Janssen F, Lub R, Bos JHJ, De Vries FM, Vansteelandt S, et al. Birth cohort appeared to confound effect estimates of guideline changes on statin utilization. J Clin Epidemiol 2015;68(3):334-340.

8. Blecker S, Goldfeld K, Park H, Radford MJ, Munson S, Francois F, et al. Impact of an Intervention to Improve Weekend Hospital Care at an Academic Medical Center: An Observational Study. Journal of General Internal Medicine 2015;30(11):1657-1664.

9. Borde JP, Litterst S, Ruhnke M, Feik R, Hübner J, deWith K, et al. Implementing an intensified antibiotic stewardship programme targeting cephalosporin and fluoroquinolone use in a 200-bed community hospital in Germany. Infection 2015;43(1):45-50.

10. Borde JP, Kern WV, Hug M, Steib-Bauert M, With Kd, Busch H, et al. Implementation of an intensified antibiotic stewardship programme targeting third-generation cephalosporin and fluoroquinolone use in an emergency medicine department. Emergency Medicine Journal 2015 July 01;32(7):509-515.

11. Boreland F, Lyle D, Brown A, Perkins D. Effectiveness of introducing point of care capillary testing and linking screening with routine appointments for increasing blood lead screening rates of young children: a before-after study. Archives of Public Health 2015;73(1):60.

12. Boyce RM, Muiru A, Reyes R, Ntaro M, Mulogo E, Matte M, et al. Impact of rapid diagnostic tests for the diagnosis and treatment of malaria at a peripheral health facility in Western Uganda: an interrupted time series analysis. Malaria Journal 2015;14:203.

13. Boyer A, Couallier V, Clouzeau B, Lasheras A, M'zali F, Kann M, et al. Control of extended-spectrum β-lactamase–producing Enterobacteriaceae nosocomial acquisition in an intensive care unit: A time series regression analysis. Am J Infect Control 2015;43(12):1296-1301.

14. Brown MJ, Kor DJ, Curry TB, Marmor Y, Rohleder TR. A Coordinated Patient Transport System for ICU Patients Requiring Surgery: Impact on Operating Room Efficiency and ICU Workflow. Journal For Healthcare Quality 2015;37(6):354-362.

15. Burden AM, Tadrous M, Calzavara A, Cadarette SM. Uptake and characteristics of zoledronic acid and denosumab patients and physicians in Ontario, Canada: impact of drug formulary access. Osteoporosis Int 2015;26(5):1525-1533.

16. Cecil E, Bottle A, Sharland M, Saxena S. Impact of UK Primary Care Policy Reforms on Short-Stay Unplanned Hospital Admissions for Children With Primary Care–Sensitive Conditions. The Annals of Family Medicine 2015 May 01;13(3):214-220.

17. Chaisson LH, Katamba A, Haguma P, Ochom E, Ayakaka I, Mugabe F, et al. Theory-Informed Interventions to Improve the Quality of Tuberculosis Evaluation at Ugandan Health Centers: A Quasi-Experimental Study. PLOS ONE 2015;10(7):e0132573.

18. Chambers EC, Wylie-Rosett J, Blank AE, Ouziel J, Hollingsworth N, Riley RW. Increasing Referrals to a YMCA-Based Diabetes Prevention Program: Effects of Electronic Referral System Modification and Provider Education in Federally Qualified Health Centers. Prev Chronic Dis 2015;12:E189.

19. Chen BK, Yang YT, Yang C. Trends in amenable deaths based on township income quartiles in Taiwan, 1971–2008: did universal health insurance close the gap? Journal of Public Health 2016;38(4):e536.

20. Cheng VCC, Chau PH, So SYC, Chen JHK, Poon RWS, Wong SCY, et al. Containment of Clostridium difficile infection without reduction in antimicrobial use in Hong Kong. European Journal of Clinical Microbiology & Infectious Diseases 2015;34(7):1381-1386.

21. Chung YK, Kim J, Lee SS, Lee J, Kim H, Shin K, et al. Effect of daily chlorhexidine bathing on acquisition of carbapenem-resistant Acinetobacter baumannii (CRAB) in the medical intensive care unit with CRAB endemicity. Am J Infect Control 2015;43(11):1171-1177.

22. Cresswell JA, Assarag B, Meski F, Filippi V, Ronsmans C. Trends in health facility deliveries and caesarean sections by wealth quintile in Morocco between 1987 and 2012. Tropical Medicine & International Health 2015;20(5):607-616.

23. Creswell J, Rai B, Wali R, Sudrungrot S, Adhikari LM, Pant R, et al. Introducing new tuberculosis diagnostics: the impact of Xpert® MTB/RIF testing on case notifications in Nepal. The International Journal of Tuberculosis and Lung Disease 2015;19(5):545-551.

24. Dayer MJ, Jones S, Prendergast B, Baddour LM, Lockhart PB, Thornhill MH. Incidence of infective endocarditis in England, 2000–13: a secular trend, interrupted time-series analysis. The Lancet 2015;385(9974):1219-1228.

25. DeGroot, J., Anderson, L., Chen, Y., Birken, C., Parkin, P., Carsley, S., Khovratovich, M., Mamdani, M., Maguire,J. Mandatory labeling requirements and over-the-counter cough and cold medication use in early childhood. Can J Public Health 2016;106(8).

26. Delcher C, Wagenaar AC, Goldberger BA, Cook RL, Maldonado-Molina MM. Abrupt decline in oxycodone-caused mortality after implementation of Florida's Prescription Drug Monitoring Program. Drug and Alcohol Dependence 2015;150:63-68.

27. Devkaran S, O’Farrell PN. The impact of hospital accreditation on quality measures: an interrupted time series analysis. BMC Health Services Research 2015;15(1):137.

28. Dinh MM, Green TC, Bein KJ, Lo S, Jones A, Johnson T. Emergency department clinical redesign, team-based care and improvements in hospital performance: A time series analysis. Emergency Medicine Australasia 2015;27(4):317-322.

29. Doernberg SB, Dudas V, Trivedi KK. Implementation of an antimicrobial stewardship program targeting residents with urinary tract infections in three community long-term care facilities: a quasi-experimental study using time-series analysis. Antimicrobial Resistance and Infection Control 2015;4:54.

30. Dunne RM, Ip IK, Abbett S, Gershanik EF, Raja AS, Hunsaker A, et al. Effect of Evidence-based Clinical Decision Support on the Use and Yield of CT Pulmonary Angiographic Imaging in Hospitalized Patients. Radiology 2015;276(1):167-174.

31. Dusetzina SB, Ellis S, Freedman RA, Conti RM, Winn AN, Chambers JD, et al. How Do Payers Respond to Regulatory Actions? The Case of Bevacizumab. JOP 2015;11(4):313-318.

32. Elliott DJ, Williams KD, Wu P, Kher HV, Michalec B, Reinbold N, et al. An Interdepartmental Care Model to Expedite Admission from the Emergency Department to the Medical ICU. Joint Commission Journal on Quality and Patient Safety 2015;41(12):542-549.

33. Estripeaut D, Contreras R, Tinajeros O, Castrejón MM, Shafi F, Ortega-Barria E, et al. Impact of Hepatitis A vaccination with a two-dose schedule in Panama: Results of epidemiological surveillance and time trend analysis. Vaccine 2015;33(28):3200-3207.

34. Fargo KL, Johnston J, Stevenson KB, Deutscher M, Reed EE. Impact of a Cost Visibility Tool in the Electronic Medical Record on Antibiotic Prescribing in an Academic Medical Center. Hosp Pharm 2015;50(6):496-504.

35. Fedeli U, Zorzi M, Urso EDL, Gennaro N, Dei Tos AP, Saugo M. Impact of fecal immunochemical test-based screening programs on proximal and distal colorectal cancer surgery rates: A natural multiple-baseline experiment. Cancer 2015;121(22):3982-3989.

36. Flett KB, Ozonoff A, Graham DA, Sandora TJ, Priebe GP. Impact of Mandatory Public Reporting of Central Line–Associated Bloodstream Infections on Blood Culture and Antibiotic Utilization in Pediatric and Neonatal Intensive Care Units. Infection Control & Hospital Epidemiology 8 00;36(8):878-885.

37. Friesen KJ, Bugden SC. The effectiveness and limitations of regulatory warnings for the safe prescribing of citalopram. Drug, Healthcare and Patient Safety 2015;7:139-145.

38. Gallagher N, Cardwell C, Hughes C, O'Reilly D. Increase in the pharmacological management of Type 2 diabetes with pay-for-performance in primary care in the UK. Diabetic Med 2015;32(1):62-68.

39. Gebrehiwot TG, San Sebastian M, Edin K, Goicolea I. The Health Extension Program and Its Association with Change in Utilization of Selected Maternal Health Services in Tigray Region, Ethiopia: A Segmented Linear Regression Analysis. PLOS ONE 2015;10(7):e0131195.

40. Giladi AM, Chung KC, Aliu O. Changes in Use of Autologous and Prosthetic Postmastectomy Reconstruction after Medicaid Expansion in New York State. Plast Reconstr Surg 2015;135(1).

41. Giladi A, Aliu O, Chung K. The Effect of Medicaid Expansion on Delivery of Finger and Thumb Replantation Care to Medicaid Beneficiaries and the Uninsured. Plastic and Reconstructive Surgery 2015 Nov;136(5):647e.

42. Green RA, Hripcsak G, Salmasian H, Lazar EJ, Bostwick SB, Bakken SR, et al. Intercepting Wrong-Patient Orders in a Computerized Provider Order Entry System. Ann Emerg Med 2015;65(6):686.e1.

43. Hacker K, Penfold R, Arsenault L, Zhang F, Soumerai SB, Wissow LS. Effect of Behavioral Health Screening and Co-located Services on Ambulatory and Inpatient Uitilization. Psychiatr Serv 2015;66(11):1141-1148.

44. Hamilton I, Lloyd C, Bland JM, Savage Grainge A. The impact of assertive outreach teams on hospital admissions for psychosis: a time series analysis. J Psychiatr Ment Health Nurs 2015;22(7):484-490.

45. Han E, Chae S, Kim N, Park S. Effects of pharmaceutical cost containment policies on doctors’ prescribing behavior: Focus on antibiotics. Health Policy 2015;119(9):1245-1254.

46. Hanatani T, Sai K, Tohkin M, Segawa K, Saito Y. Impact of Japanese regulatory action on metformin-associated lactic acidosis in type II diabetes patients. International Journal of Clinical Pharmacy 2015;37(3):537-545.

47. Haren MT, Setchell J, John DL, Daniel M. The impacts of withdrawal and replacement of general practitioner services on aeromedical service trends: a 13-year interrupted time-series study in Tennant Creek, Northern Territory. BMC Health Services Research 2015;15(1):456.

48. Hartung DM, Middleton L, Markwardt S, Williamson K, Ketchum K. Changes in Long-acting β-agonist Utilization After the FDA’s 2010 Drug Safety Communication. Clin Ther 2015;37(1):123.e1.

49. Hernandez-Santiago V, Marwick CA, Patton A, Davey PG, Donnan PT, Guthrie B. Time series analysis of the impact of an intervention in Tayside, Scotland to reduce primary care broad-spectrum antimicrobial use. Journal of Antimicrobial Chemotherapy 2015 August 01;70(8):2397-2404.

50. Hsu JC, Cheng Ching-Lan, Ross-Degnan Dennis, Wagner AK, Fang Z, Kao YY, et al. Effects of safety warnings and risk management plan for Thiazolidinediones in Taiwan. Pharmacoepidemiol Drug Saf 2015;24(10):1026-1035.

51. Hsu JC, Ross-Degnan D, Wagner AK, Cheng C, Yang YK, Zhang F, et al. Utilization of oral antidiabetic medications in Taiwan following strategies to promote access to medicines for chronic diseases in community pharmacies. Journal of Pharmaceutical Policy and Practice 2015;8(1):15.

52. Hsu JC, Ross-Degnan D, Wagner AK, Zhang F, Lu CY. How Did Multiple FDA Actions Affect the Utilization and Reimbursed Costs of Thiazolidinediones in US Medicaid? Clin Ther 2015;37(7):1432.e1.

53. Hutcheon JA, Strumpf EC, Harper S, Giesbrecht E. Maternal and neonatal outcomes after implementation of a hospital policy to limit low-risk planned caesarean deliveries before 39 weeks of gestation: an interrupted time-series analysis. BJOG: An International Journal of Obstetrics & Gynaecology 2015;122(9):1200-1206.

54. Hutchinson AF, Parikh S, Tacey M, Harvey PA, Lim WK. A longitudinal cohort study evaluating the impact of a geriatrician-led residential care outreach service on acute healthcare utilisation. Age and Ageing 2015 May 01;44(3):365-370.

55. Ir P, Korachais C, Chheng K, Horemans D, Van Damme W, Meessen B. Boosting facility deliveries with results-based financing: a mixed-methods evaluation of the government midwifery incentive scheme in Cambodia. BMC Pregnancy and Childbirth 2015;15(1):170.

56. Jain S, Frank G, McCormick K, Wu B, Johnson BA. Impact of Physician Scorecards on Emergency Department Resource Use, Quality, and Efficiency. Pediatrics 2015;136(3):e670.

57. Jenkins TC, Knepper BC, Shihadeh K, Haas MK, Sabel AL, Steele AW, et al. Long-Term Outcomes of an Antimicrobial Stewardship Program Implemented in a Hospital with Low Baseline Antibiotic Use. Infection control and hospital epidemiology 2015;36(6):664-672.

58. Jiang M, Hughes DR, Duszak R. Screening Mammography Rates in the Medicare Population before and after the 2009 U.S. Preventive Services Task Force Guideline Change: An Interrupted Time Series Analysis. Women's Health Issues 2015;25(3):239-245.

59. Jones SL, Ashton CM, Kiehne L, Gigliotti E, Bell-Gordon C, Disbot M, et al. Reductions in Sepsis Mortality and Costs After Design and Implementation of a Nurse-Based Early Recognition and Response Program. Joint Commission journal on quality and patient safety / Joint Commission Resources 2015;41(11):483-491.

60. Judge A, Wallace G, Prieto-Alhambra D, Arden NK, Edwards CJ. Can the publication of guidelines change the management of early rheumatoid arthritis? An interrupted time series analysis from the United Kingdom. Rheumatology 2015 December 01;54(12):2244-2248.

61. Karkouti K, McCluskey SA, Callum J, Freedman J, Selby R, Timoumi T, et al. Evaluation of a Novel Transfusion Algorithm Employing Point-of-care Coagulation Assays in Cardiac SurgeryA Retrospective Cohort Study with Interrupted Time–Series Analysis. Anesthesiology 2015;122(3):560-570.

62. Kendrick T, Stuart B, Newell C, Geraghty AWA, Moore M. Did NICE guidelines and the Quality Outcomes Framework change GP antidepressant prescribing in England? Observational study with time trend analyses 2003–2013. J Affect Disord 2015;186:171-177.

63. Kim SH, Cho BL, Shin DW, Hwang S, Lee H, Ahn EM, et al. The Effect of Asthma Clinical Guideline for Adults on Inhaled Corticosteroids PrescriptionTrend: A Quasi-Experimental Study. J Korean Med Sci 2015;30(8):1048-1054.

64. Kim Y, Kim J. Impact of a financial incentive policy on Korean nurse staffing. Int Nurs Rev 2015;62(2):171-179.

65. Lal S, Ndyomugenyi R, Alexander ND, Lagarde M, Paintain L, Magnussen P, et al. Health Facility Utilisation Changes during the Introduction of Community Case Management of Malaria in South Western Uganda: An Interrupted Time Series Approach. PLOS ONE 2015;10(9):e0137448.

66. Lawes T, Lopez-Lozano J, Nebot CA, Macartney G, Subbarao-Sharma R, Dare CR, et al. Effects of national antibiotic stewardship and infection control strategies on hospital-associated and community-associated meticillin-resistant Staphylococcus aureus infections across a region of Scotland: a non-linear time-series study. The Lancet Infectious Diseases 2015;15(12):1438-1449.

67. Li S, Dor A. How Do Hospitals Respond to Market Entry? Evidence from a Deregulated Market for Cardiac Revascularization. Health Econ 2015;24(8):990-1008.

68. Liu C, Zhang X, Wan J. Public reporting influences antibiotic and injection prescription in primary care: a segmented regression analysis. J Eval Clin Pract 2015;21(4):597-603.

69. Lobos A-. Routine Medical Emergency Team Assessments of Patients Discharged from the PICU: Description of a Medical Emergency Team Follow-Up Program. Pediatric critical care medicine 2015;16(4):359-365.

70. Lowthian J, Curtis A, Straney L, McKimm A, Keogh M, Stripp A. Redesigning emergency patient flow with timely quality care at the Alfred. Emergency Medicine Australasia 2015;27(1):35-41.

71. Lu CY, Zhang F, Lakoma MD, Butler MG, Fung V, Larkin EK, et al. Asthma Treatments and Mental Health Visits After a Food and Drug Administration Label Change for Leukotriene Inhibitors. Clin Ther 2015;37(6):1280-1291.

72. Mahant S, Hall M, Ishman SL, Morse R, Mittal V, Mussman GM, et al. Association of National Guidelines With Tonsillectomy Perioperative Care and Outcomes. Pediatrics 2015;136(1):53-60.

73. Martin BI, Lurie JD, Tosteson ANA, Deyo RA, Farrokhi FR, Mirza SK. Use of bone morphogenetic protein among patients undergoing fusion for degenerative diagnoses in the United States, 2002 to 2012. The Spine Journal 2015;15(4):692-699.

74. Marufu O, Desai N, Aldred D, Brown T, Eltringham I. Analysis of interventions to reduce the incidence of Clostridium difficile infection at a London teaching hospital trust, 2003‒2011. J Hosp Infect 2015;89(1):38-45.

75. McGinty EE, Busch SH, Stuart EA, Huskamp HA, Gibson TB, Goldman HH, et al. Federal Parity Law Associated With Increased Probability Of Using Out-Of-Network Substance Use Disorder Treatment Services. Health Aff (Millwood ) 2015;34(8):1331-1339.

76. McIlroy G, Thomas SK, Coleman JJ. Second-generation antipsychotic drug use in hospital inpatients with dementia: the impact of a safety warning on rates of prescribing. Journal of Public Health 2015 June 01;37(2):346-352.

77. McKenna B, Furness T, Brown S, Tacey M, Hiam A, Wise M. Police and clinician diversion of people in mental health crisis from the Emergency Department: a trend analysis and cross comparison study. BMC Emergency Medicine 2015;15(1):14.

78. Mellou K, Sideroglou T, Papaevangelou V, Katsiaflaka A, Bitsolas N, Verykouki E, et al. Considerations on the Current Universal Vaccination Policy against Hepatitis A in Greece after Recent Outbreaks. PLOS ONE 2015;10(1):e0116939.

79. Milder EA, Rizzi MD, Morales KH, Ross RK, Lautenbach E,Gerber JS. Impact of a new practice guideline on antibiotic use with pediatric tonsillectomy. JAMA Otolaryngology–Head & Neck Surgery 2015 May 1;141(5):410-416.

80. Narayan H, Thomas SH, Eddleston M, Dear JW, Sandilands E, Nicholas Bateman D. Disproportionate effect on child admissions of the change in Medicines and Healthcare Products Regulatory Agency guidance for management of paracetamol poisoning: an analysis of hospital admissions for paracetamol overdose in England and Scotland. Br J Clin Pharmacol 2015;80(6):1458-1463.

81. Newitt S, Myles PR, Birkin JA, Maskell V, Slack RCB, Nguyen-Van-Tam JS, et al. Impact of infection control interventions on rates of Staphylococcus aureus bacteraemia in National Health Service acute hospitals, East Midlands, UK, using interrupted time-series analysis. J Hosp Infect 2015;90(1):28-37.

82. Ng W, Brown A, Alexander D, Ho MF, Kerr B, Amato M, et al. A multifaceted prevention program to reduce infection after cesarean section: Interventions assessed using an intensive postdischarge surveillance system. Am J Infect Control 2015;43(8):805-809.

83. Nirmeen S, Dalia D, Adel A, Natalia H, Darrin B. Evaluation of a protocol-based intervention to promote timely switching from intravenous to oral paracetamol for post-operative pain management: an interrupted time series analysis. J Eval Clin Pract 2015;21(6):1081-1088.

84. Overman RA, Farley JF, Curtis JR, Zhang J, Gourlay ML, Deal CL. DXA Utilization Between 2006 and 2012 in Commercially Insured Younger Postmenopausal Women. Journal of Clinical Densitometry 2015;18(2):145-149.

85. Owusu-Edusei Jr. K, Carroll DS, Gift TL. Examining Fluoroquinolone Claims Among Gonorrhea-Associated Prescription Drug Claims, 2000–2010. Am J Prev Med 2015;49(5):761-764.

86. Pant S, Patel NJ, Deshmukh A, Golwala H, Patel N, Badheka A, et al. Trends in Infective Endocarditis Incidence, Microbiology, and Valve Replacement in the United States From 2000 to 2011. J Am Coll Cardiol 2015;65(19):2070-2076.

87. Park J, Lee N, Cho Y, Yang Y. Modified constraint-induced movement therapy for clients with chronic stroke: interrupted time series (ITS) design. Journal of Physical Therapy Science 2014;27(3):963-966.

88. Petrou P. The Ariadne's thread in co-payment, primary health care usage and financial crisis: findings from Cyprus public health care sector. Public Health 2015;129(11):1503-1509.

89. Petrou P. An Interrupted Time-Series Analysis to Assess Impact of Introduction of Co-Payment on Emergency Room Visits in Cyprus. Applied Health Economics and Health Policy 2015;13(5):515-523.

90. Pow JL, Baumeister AA, Hawkins MF, Cohen AS, Garand JC. Deinstitutionalization of American Public Hospitals for the Mentally Ill Before and After the Introduction of Antipsychotic Medications. Harv Rev Psychiatry 2015;23(3).

91. Redd V, Levin S, Toerper M, Creel A, Peterson S. Effects of Fully Accessible Magnetic Resonance Imaging in the Emergency Department. Acad Emerg Med 2015;22(6):741-749.

92. Roifman I, Rezai MR, Wijeysundera HC, Chow BJW, Wright GA, Tu JV. Utilization of cardiac computed tomography angiography and outpatient invasive coronary angiography in Ontario, Canada. Journal of Cardiovascular Computed Tomography 2015;9(6):567-571.

93. Rosenstein MG, Nijagal M, Nakagawa S, Gregorich SE, Kuppermann M. The Association of Expanded Access to a Collaborative Midwifery and Laborist Model With Cesarean Delivery Rates. Obstet Gynecol 2015;126(4):716-723.

94. Sarma JB, Marshall B, Cleeve V, Tate D, Oswald T, Woolfrey S. Effects of fluoroquinolone restriction (from 2007 to 2012) on resistance in Enterobacteriaceae: interrupted time-series analysis. J Hosp Infect 2015;91(1):68-73.

95. Sarma JB, Marshall B, Cleeve V, Tate D, Oswald T, Woolfrey S. Effects of fluoroquinolone restriction (from 2007 to 2012) on Clostridium difficile infections: interrupted time-series analysis. Journal of Hospital Infection 2015;91(1):74-80.

96. Saxena S, Atchison C, Cecil E, Sharland M, Koshy E, Bottle A. Additive impact of pneumococcal conjugate vaccines on pneumonia and empyema hospital admissions in England. J Infect 2015;71(4):428-436.

97. Schaffer AL, Buckley NA, Dobbins TA, Banks E, Pearson S-. The crux of the matter: Did the ABC's catalyst program change Statin use in Australia? Medical Journal of Australia 2015;202(11):591-595.

98. Schoenfeld AJ, Weaver MJ, Power RK, Harris MB. Does Health Reform Change Femoral Neck Fracture Care? A Natural Experiment in the State of Massachusetts. J Orthop Trauma 2015;29(11).

99. Simpao AF, Ahumada LM, Desai BR, Bonafide CP, Gálvez JA, Rehman MA, et al. Optimization of drug–drug interaction alert rules in a pediatric hospital's electronic health record system using a visual analytics dashboard. Journal of the American Medical Informatics Association 2015;22(2):361-369.

100. Smith JJ, Johnston JM, Hiratsuka VY, Dillard DA, Tierney S, Driscoll DL. Medical home implementation and trends in diabetes quality measures for AN/AI primary care patients. Primary Care Diabetes 2015;9(2):120-126.

101. Sood MM, Akbari A, Hiebert B, Hiremath S, Komenda P, Rigatto C, et al. Trends in Arteriovenous Fistula Use at Dialysis Initiation After Automated eGFR Reporting. Semin Dial 2015;28(4):439-445.

102. Stock D, Rabeneck L, Baxter NN, Paszat LF, Sutradhar R, Yun L, et al. Mailed participant reminders are associated with improved colonoscopy uptake after a positive FOBT result in Ontario’s ColonCancerCheck program. Implementation Science : IS 2015;10:35.

103. Sánchez DP, Guillén JJ, Torres AM, Arense JJ, López Á, Sánchez FI. La recuperación del consumo farmacéutico tras la modificación del copago: evidencia de un servicio regional de salud. Atención Primaria 2015;47(7):411-418.

104. Talbot TR, Carr D, Lee Parmley C, Martin BJ, Gray B, Ambrose A, et al. Sustained Reduction of Ventilator-Associated Pneumonia Rates Using Real-Time Course Correction With a Ventilator Bundle Compliance Dashboard. Infection Control & Hospital Epidemiology 2015;36(11):1261-1267.

105. Tippett EC, Chen BK. Association of Attorney Advertising and FDA Action with Prescription Claims: A Time Series Segmented Regression Analysis. Drug Safety 2015;38(12):1169-1178.

106. Trifirò G, Parrino F, Sultana J, Giorgianni F, Ferrajolo C, Bianchini E, et al. Drug Interactions with Levothyroxine Therapy in Patients with Hypothyroidism: Observational Study in General Practice. Clinical Drug Investigation 2015;35(3):187-195.

107. Tung Y, Chang G, Cheng S. Long-Term Effect of Fee-For-Service–Based Reimbursement Cuts on Processes and Outcomes of Care for Stroke. Circ Cardiovasc Qual Outcomes 2015;8(1):30-37.

108. Uijtendaal EV, Zwart-van Rijkom, Jeannette E F, de Lange DW, Lalmohamed A, van Solinge WW, Egberts TCG. Influence of a strict glucose protocol on serum potassium and glucose concentrations and their association with mortality in intensive care patients. Critical Care 2015;19(1):270.

109. Valerio M, Muñoz P, Rodríguez CG, Caliz B, Padilla B, Fernández-Cruz A, et al. Antifungal stewardship in a tertiary-care institution: a bedside intervention. Clinical Microbiology and Infection 2015;21(5):492.e9.

110. Viale P, Tumietto F, Giannella M, Bartoletti M, Tedeschi S, Ambretti S, et al. Impact of a hospital-wide multifaceted programme for reducing carbapenem-resistant Enterobacteriaceae infections in a large teaching hospital in northern Italy. Clinical Microbiology and Infection 2015;21(3):242-247.

111. Vuković M, Gvozdenović BS, Ranković M, McCormick BP, Vuković DD, Gvozdenović BD, et al. Can Didactic Continuing Education Improve Clinical Decision Making and Reduce Cost of Quality? Evidence From a Case Study. J Contin Educ Health Prof 2015;35(2):109-118.

112. Wang Z, Bhattacharyya T. Trends of non-union and prescriptions for non-steroidal anti-inflammatory drugs in the United States, 1993–2012. Acta Orthopaedica 2015;86(5):632-637.

113. Waters TM, Daniels MJ, Bazzoli GJ,et al. Effect of medicare’s nonpayment for hospital-acquired conditions: Lessons for future policy. JAMA Internal Medicine 2015 March 1;175(3):347-354.

114. Williams DJ, Edwards KM, Self WH, Zhu Y, Ampofo K, Pavia AT, et al. Antibiotic Choice for Children Hospitalized With Pneumonia and Adherence to National Guidelines. Pediatrics 2015.

115. Yang J, Kim M, Park Y, Lee E, Jung CY, Kim S. The effect of the introduction of a nationwide DUR system where local DUR systems are operating—The Korean experience. Int J Med Inf 2015;84(11):912-919.

116. Yoo K, Lee SG, Park S, Kim TH, Ahn J, Cho M, et al. Effects of drug price reduction and prescribing restrictions on expenditures and utilisation of antihypertensive drugs in Korea. BMJ Open 2015 July 01;5(7).
